# The Value of Reference Genomes in the Conservation of Threatened Species

**DOI:** 10.3390/genes10110846

**Published:** 2019-10-25

**Authors:** Parice Brandies, Emma Peel, Carolyn J. Hogg, Katherine Belov

**Affiliations:** School of Life & Environmental Sciences, The University of Sydney, Sydney 2006, Australia; parice.brandies@sydney.edu.au (P.B.); emma.peel@sydney.edu.au (E.P.); carolyn.hogg@sydney.edu.au (C.J.H.)

**Keywords:** conservation, genomes, Tasmanian devil

## Abstract

Conservation initiatives are now more crucial than ever—over a million plant and animal species are at risk of extinction over the coming decades. The genetic management of threatened species held in insurance programs is recommended; however, few are taking advantage of the full range of genomic technologies available today. Less than 1% of the 13505 species currently listed as threated by the International Union for Conservation of Nature (IUCN) have a published genome. While there has been much discussion in the literature about the importance of genomics for conservation, there are limited examples of how having a reference genome has changed conservation management practice. The Tasmanian devil (*Sarcophilus harrisii*), is an endangered Australian marsupial, threatened by an infectious clonal cancer devil facial tumor disease (DFTD). Populations have declined by 80% since the disease was first recorded in 1996. A reference genome for this species was published in 2012 and has been crucial for understanding DFTD and the management of the species in the wild. Here we use the Tasmanian devil as an example of how a reference genome has influenced management actions in the conservation of a species.

## 1. Introduction

We are currently in the midst of a global sixth mass extinction event, with biodiversity rapidly declining around the world [1], and extinction rates are accelerating [2]. Australia has the worst mammal extinction rate of any country, with 25 mammals declared extinct since European settlement and almost 20% of current mammalian species listed as vulnerable [2,3,4,5]. This significant decline is concerning as Australia is one of seventeen “megadiverse” countries that comprises a large proportion of the Earth’s biological diversity [6]. Megadiverse countries have at least 5,000 endemic plant species and have marine ecosystems within its borders [6]. In addition to this, 87% of Australian mammals, 93% of Australian reptiles, and 94% of Australian frogs are endemic to Australia [7]. Therefore, conservation initiatives that protect and maintain Australia’s biodiversity are now more crucial than ever.

Only 39% of the 1,890 Australian species (517 animals; 1373 plants), listed as threatened under the Environment Protection and Biodiversity Conservation Act (EPBC Act), have a recovery plan in place to improve their threat status [8]. These recovery plans set out management and research actions to slow population decline and promote recovery of threatened species and communities. This is achieved by providing a framework for key interest groups and government agencies to coordinate their efforts to improve the plight of threatened species [8]. Management actions range from mitigating threatening processes such as predation, habitat loss, or change, in addition to research into basic species biology, ecosystem integration, and genetics. The main goal of recovery plans is to maintain the long-term viability of a chosen population/community. Maintaining genetic diversity is an important component of population viability as it assists with mitigating negative effects associated with inbreeding and arms populations with the potential to adapt to future environmental change [9,10,11]. As such, understanding a populations’ inherent genetic diversity, in addition to their historical diversity and future potential, is of utmost importance in species conservation. For this reason, more than 80% of the current 200 Australian national vertebrate recovery plans have some form of genetic action listed in the species’ recovery plan. Yet, less than 15% of these recovery plans have any form of genetic or genomic data available, either in existence or currently in development. Here we refer to genetic data as information based on specific, limited regions of the genome (e.g., targeted gene sequencing, microsatellite analysis, etc.), whilst genomic data is information based on the whole genome (e.g., whole genome sequencing/resequencing, whole-genome single nucleotide polymorphism (SNP) analysis/reduced representation sequencing, etc.).

Advances in sequencing technologies and the reduction in sequencing costs have given rise to the era of genomics, whereby holistic genome-wide approaches are rapidly replacing traditional genetic marker approaches in many non-model species [12,13,14]. Although recent reviews have highlighted the importance of implementing genomic data into conservation initiatives [13,15,16], the application of such powerful advances in sequencing technologies is lacking in the current literature. This limited use in conservation may be due to a number of reasons including: costs, a lack of understanding of the potential of new genomics approaches, lack of expertise in developing and utilizing the data, and the absence of a reference genome for the species of interest (or a closely-related species) [13,15,17]. The latter is an important concern as the generation of a reference genome requires considerable expertise, funds, computational resources, and time that are not often accessible by wildlife managers and conservation teams [15,18].

Of the 13505 animal species that are listed as threatened (Lower Risk/Conservation Dependent or worse) on the International Union for Conservation of Nature (IUCN) Red List [2], 108 (< 1%) have published genomes on NCBI [19]. This equates to only 6% of the 1842 animal genomes currently available on NCBI [19]. Creating high-quality reference genomes that can provide insights into species evolution and biology is a costly task (~$30,000 for an average eukaryotic genome size of 2.5 Gbp [20]), and also requires large collaborative groups to provide expertise from varying fields (e.g., [21,22,23]). Fortunately, in recent years a number of national and international consortia and genome projects have been formed with the aim of creating high-quality reference genomes for species spanning the phylogenetic tree of life including: the Earth Biogenome Project (EBP) [20], the Genome 10K Project (G10K) [24,25], the Vertebrate Genomes Project (VGP) [26], the Bird 10K Project (B10K) [27], the Bat 1K Project (Bat1K) [28], the Global Invertebrate Genomics Alliance (GIGA) [29,30], and the Oz Mammal Genomics initiative (OMG) [31], to name a few. The goal of many of these consortia is to bring together the required expertise to generate reference genomes of a sufficient quality, which are publicly available to the science community, thereby providing the vital resources required to implement genomics into conservation management better [13,15,18]. However, just providing the reference genomes or genomic data is not enough to improve conservation outcomes. Geneticists need to continually communicate how genomic techniques can be utilized in a cost-effective manner to assist species conservation better [17,32]. As highlighted by Taylor et al. [33], targeted education and training is also required to teach conservation managers how to interpret and utilize genomic data. To better assist conservation managers, a number of groups and communities have already been established to assist in providing conservation genetics advice for threated species management. These include the IUCN/SSC (Species Survival Commission) Conservation Genetics Specialist Group (CGSG), the Genetic Composition Working Group of GEO BON (Group on Earth Observations Biodiversity Observation Network), and the pan-European COST (Cooperation in Science and Technology) action ConGRESS (Conservation Genetic Resources for Effective Species Survival) (for further information and examples from these groups, see Holderegger et al. [34]). Conservationists in their respective countries can get in touch with these groups to obtain the contact details of geneticists who work in their region who may be able to assist them with their management needs.

While a number of papers have reviewed current genomic techniques and the way they can, or have been, applied to assist in conservation decisions across species [15,17], questions are still raised as to whether reference genomes are necessary for species conservation. Reference genomes hold the key to investigate a number of paradigms that are essential for species conservation, including: demography, inbreeding, hybridization, disease susceptibility, behavioral ecology, and adaptation [12,13,15,16,18]. Here we demonstrate the value of a reference genome to the conservation effort of an endangered species, the Tasmanian devil (*Sarcophilus harrisii*), and how this information has been applied in real-time management practice [35].

The Tasmanian devil, an endangered Australian marsupial, is often used in the literature as an example of how genetics/genomics approaches could be used in conservation [12,13,36]. However, something that is not often discussed is that having a reference genome for this species is one of the key factors that contributed to using genomics in management practice. Although this species has a unique conservation issue, low genetic diversity coupled with an infectious clonal cancer, the methods described herein apply to many other threatened species. Here we show how the reference genome has allowed a range of conservation questions to be answered in a timely, cost-effective manner and enabled conservation researchers to adapt to the rapid advances in genomic technologies.

## 2. The Tasmanian Devil and Its Genome 

The Tasmanian devil is the largest extant carnivorous marsupial, native to mainland Tasmania, Australia. The emergence of transmissible cancer, devil facial tumor disease (DFTD) in the mid-1990s has led to a rapid population decline of up to 80% across their range [37]. In 2003, the Tasmanian and Australian governments responded to the disease threat by establishing the Save the Tasmanian Devil Program (STDP). Since then, researchers, wildlife managers, and the zoo industry have worked closely with the STDP to ensure that Tasmanian devils have a sustainable ecological function in the Tasmanian ecosystem and landscape [35,38]. This work has included a range of activities such as monitoring of wild populations, developing an insurance population, describing and characterizing the disease, and developing new genomic tools to understand the disease and the Tasmanian devil [38]. 

Prior to the publication of a reference genome for the Tasmanian devil, traditional genetic approaches such as MHC (major histocompatibility complex) typing and microsatellite analysis were used to explore genetic diversity at specific genes as well as general genetic diversity in the species [39,40,41]. These techniques were able to show that the Tasmanian devil had low genetic diversity [39,40,41,42]. However, the low rates of polymorphism for most of these markers did not have high enough resolution to assist in answering crucial conservation questions such as determining founder relatedness within the insurance population [43,44], identifying high-resolution population substructure [45], or to better understand the origin and evolution of DFTD [46]. In instances such as these, further genomic data was required to improve resolution. For other threatened species, where there may be moderate to high genome-wide diversity, microsatellite markers may be highly polymorphic, and so these markers have value as a continuing genetic management tool.

To overcome this knowledge gap, the Tasmanian devil genome was sequenced independently by two different research groups in 2011 [45,46]. Miller et al. [45] sequenced the nuclear genome of two individuals (originating from extreme northwest and southeast Tasmania), as well as the tumor from one individual, using both Roche and Illumina sequencing platforms. The analysis of genome-wide SNPs confirmed low genetic diversity across the Tasmanian devil genome, as well as enabling the construction of genotyping arrays, which revealed a new population substructure and the identification of tumor-specific SNPs. However, the low contiguity of this reference genome assembly (148,891 scaffolds, scaffold N50 147 kb) limited the applicability of the data in downstream research. In 2012, a more contiguous, annotated nuclear genome (35974 scaffolds, scaffold N50 1.85 Mb), and tumor genome was published by Murchison et al. [46], resulting in the primary reference genome used today. This higher quality assembly facilitated an enormous effort in downstream genetic and genomic research. It should be noted that as of August 2019, the 2012 Tasmanian devil reference genome paper [46] has been cited over 200 times (Google Scholar Citation Search), highlighting the value of this reference genome to the research community. It is not possible to cover all of the research that has stemmed from the sequencing of the 2012 genome here. Rather, here, we present key examples of how having a reference genome has contributed to conservation decisions and outcomes for the Tasmanian devil. We also note that at the time of this publication, an updated Tasmanian devil genome assembly has been released [47]. This assembly utilized an in vitro proximity ligation technique to further improve the scaffolding of the 2012 assembly (10010 scaffolds, N50 7.75 Mb); however, chromosome assignment and annotation have not been performed at this stage. 

## 3. Conservation Applications as a Result of a Reference Genome

### 3.1. Basic Conservation Management

#### 3.1.1. Microsatellite Analysis

Traditionally population genetic measures to answer basic questions regarding population structure, population size, population dynamics (migration, bottlenecks), kinship, inbreeding, etc. [14,48] have used microsatellites, or short tandem repeats [48]. Where microsatellite markers have already been developed for the species of interest, or in a closely related species that may carry similar markers, they provide a cost-effective, quick conservation management tool [48,49]. However, for those species where appropriate microsatellite markers are not currently available, or cross-species microsatellite amplification is not effective, and a reference genome is also not available, considerable time and resources are required to develop species-specific microsatellite markers. For example, prior to sequencing the Tasmanian devil genome, 11 putatively neutral microsatellite markers were developed to assess genetic diversity in Tasmanian devils [39]. The development of these microsatellites involved the creation and screening of a genomic library, sequencing of positive clones, primer design, and PCR optimization [39]. Several years later, MHC-linked microsatellite markers were developed in a similar manner as a cheaper and faster method of investigating MHC diversity when compared to traditional MHC typing techniques, such as cloning and sequencing particular MHC regions [41]. This traditional microsatellite isolation and the marker development approaches require considerable laboratory expertise, time, and funds [49], that today may be better spent developing more powerful molecular approaches (see Reduced Representation Sequencing section below). 

Contrarily, the availability of the Tasmanian devil reference genome enabled 22 additional microsatellite markers to be identified and developed in a much faster, cost-effective manner using bioinformatic methods [50]. More importantly, each of these microsatellites were known to be in non-coding regions across all of the autosomes, providing a greater representation of neutral genome-wide diversity in comparison to the original 11 putatively neutral microsatellites. It has previously been estimated that the development of just 10 microsatellite markers without prior genetic data can cost up to $10000 [51]. The availability of a reference genome mitigates the need for traditional microsatellite isolation procedures, and therefore, significantly reduces costs associated with marker development (< $1000 for primer optimization and testing). Additionally, the commercial development of microsatellite-based PCR kits resulted in further reductions in the time and cost associated with microsatellite marker development and use [50]. To date 33 microsatellite markers have successfully been applied to Tasmanian devil conservation to investigate inbreeding [50], reconstruct the pedigree of offspring born in group housing and on Maria Island [50,52,53,54], and investigate mate choice within captivity and the wild [55] (Table 1). These microsatellite markers have also successfully been applied to genotype individuals using non-invasive scat samples [56], which are notoriously known for producing low quantities of low-quality DNA [57]. Globally, microsatellite markers continue to be an effective tool in conservation decision making by answering population questions [58,59,60,61,62]. They are particularly valuable when using non-invasive samples that are often unsuitable for more complex genomic methods that require high-quality input DNA, such as reduced representation sequencing and other whole-genome sequencing methods [15]. A reference genome allows for fast, easy, and inexpensive development of such markers, improving their utility in the conservation management space.

#### 3.1.2. Reduced Representation Sequencing

While microsatellite analysis is one of the most common population genetics tools, sometimes more statistical power is needed to address specific conservation management questions, particularly in species with low genetic diversity [43,80,81]. For instance, in the Tasmanian devil, microsatellite analysis was unable to accurately estimate the relatedness of founders sourced for the insurance population between 2006 and 2008 [43]. Single nucleotide polymorphisms (SNPs) enable greater resolution for addressing some common conservation issues such as resolving parentage and population structure, understanding genetic diversity, and identifying regions of the genome, which may be linked to important phenotypes [42]. When compared to a microsatellite approach, only 3–8 biallelic SNPs are required to be as informative as one microsatellite marker [82,83]. Reduced representation sequencing (RRS) is a simple, cost-effective approach for generating genome-wide SNP data and is gaining popularity in the conservation sector [15,42,84]. RRS relies on high-throughput sequencing of fragments generated by restriction enzyme digestion of the genome and can, therefore, easily be applied in any species. There are a variety of RRS methods currently available, including traditional RADseq [85], ddRAD [86], DArTseq [87], and others [42]. 

Both DArTseq and RADseq have been employed to collect RRS data from over 1,000 Tasmanian devils from the insurance population, Maria island and a number of wild sites [76,77,84,88,89]. RRS methods have shown to be superior in accurately estimating diversity and inferring genome-wide heterozygosity compared with microsatellite analysis and other targeted techniques [89]. Although this approach does not require a reference genome for development and use, coupling RRS data with a reference genome is advantageous in that it: i) improves the reliability of genotype calls [90]; ii) reduces the required coverage for accurate genotyping [91]; iii) provides for a greater number of SNPs [92]; iv) improves downstream population genetic inferences [92]; v) allows for SNP annotation with gene information [93]; and vi) provides the ability to compare results from differing RRS methods which are particularly important when different methods are used across time for endangered species. 

Using a reference genome guided approach in the Tasmanian devil enabled 2060 SNPs to be identified [84] much more quickly than a de novo approach. Aligning the RRS data to the reference genome provides the ability to identify genes which may be targets of future analysis, and to separate functional vs. non-functional genome diversity which could have conservation implications [94]. For example, the reference genome was able to identify candidate genes within a genomic region that displayed signatures of selection in RRS data [76], and to identify cancer-resistance candidate genes from phenotype association tests of RRS data [77] (Table 1). A number of non-synonymous SNPs have also been identified within particular genes, which have the potential to impact phenotype. Furthermore, reference alignment allows SNPs from alternative RRS datasets to be compared and combined, such as the DArTseq and RADseq data, which are important for reusing previous investments of limited conservation dollars. Recent work investigating New Zealand threatened bird species also showed the benefits of calling SNPs against conordinal, confamilial, cogeneric, and conspecific reference genomes [95]. This highlights that not every threatened species requires a reference genome, although the quality of the SNP data reduces as you move away from the genus and family level. 

### 3.2. Further Species-Specific Applications

#### 3.2.1. Reference Gene Characterization

A valuable advantage of having access to a reference genome is the ability to characterize particular genes, or gene families, that are relevant to species-specific conservation [23]. Gene characterisation is often undertaken in two main ways: in-depth, manual characterization of a specific set of genes of interest, and automatic, whole-genome annotation. The latter is achieved in two main stages: the computational phase and the annotation phase [96,97]. During the computational phase, initial gene predictions are based on several lines of evidence including transcriptome and protein data from the species of interest and several closely-related, or well-annotated species [96,97]. During the annotation phase, the most representative gene predictions (defined by the annotation pipeline) are synthesized into the final gene annotations [96,97]. The whole-genome annotation of the Tasmanian devil reference genome was achieved using the Ensemble genome annotation pipeline [46,98,99]. This automatic annotation of 18775 protein-coding genes was critical to the development of targeted SNP panels to explore diversity at important immune genes in the Tasmanian devil [69,70,71] (see SNP Panel section below), and in the identification of genes that may be linked to DFTD [46,76,77,78,100] (Table 1).

While modern-day tools, such as trainable automated gene prediction algorithms, have increased the feasibility of genome annotation of newly sequenced species within individual research groups, complete genome annotation still requires considerable bioinformatics expertise [96,97]. Manual annotation of a subset of target genes is often required. This is particularly relevant for genes that have experienced duplications and are, therefore, often unable to be automatically annotated [23,96]. In the Tasmanian devil, this was true for a number of gene families, including the Major Histocompatibility Complex (MHC), toll-like receptors (TLR), natural killer (NK) receptors, cathelicidins, behavior, and reproductive genes which were all manually annotated [69,72,75,101,102]. Annotation of these genes was essential in facilitating species-specific downstream research and informing conservation management decisions in the Tasmanian devil, such as genetic variation analyses [69,70,72,75]; selection of individuals for release to the wild [63], individuals response to the immunotherapy [66]; changes of immune function with the onset of puberty [73]; and the influence of age and DFTD on immune function [74] (Table 1). This highlights the potential of a reference genome for exploratory analysis of gene families involved in key biological processes of threatened species such as immunity, reproduction, and behavior. 

#### 3.2.2. Targeted SNP Panels

Targeted SNP panels enable diversity at particular genes to be investigated based on current conservation concerns/questions [103]. In the Tasmanian devil, an SNP panel targeting immune, behavioral, and putatively neutral loci was developed and used to genotype over 300 individuals in the insurance population [71]. This involved low-coverage resequencing of a number of individuals (see the Whole-Genome Resequencing section below), alignment of data to the reference genome, identification of target SNPs, primer design, pilot sequencing, and final genotyping. The SNP panel resolved parentage with higher confidence than microsatellite markers and also provided representative measures of genetic diversity at both functional and non-functional loci [71]. Development of another SNP panel, which targeted a range of immune genes, showed considerably low immune diversity in the species [70], which has led to further research into ways of breeding Tasmanian devils to improve genome-wide heterozygosity and functional diversity [67,68]. The Tasmanian devil reference genome was essential for aligning sequencing data and target SNP discovery allowing for management decisions to be based on both genome-wide and functional diversity (Table 1). Although custom SNP panel development can be expensive and is not simple, once developed it provides fast, accurate measures of diversity at particular genes, or genome regions, across a large number of individuals [71,104,105]. 

#### 3.2.3. Whole-Genome Resequencing

Whole-genome resequencing (WGR) involves sequencing the genome of several individuals to a predetermined level of coverage (usually between 2× and 60×) and aligning this data to an available reference genome (for examples in non-model species, see Fuentes-Pardo and Ruzzante [15]). A major application of whole-genome resequencing (WGR) is the identification of variation throughout the genome, enabling the development of more targeted approaches that can be used to explore diversity at key regions in a larger cohort of individuals [70,71]. The Tasmanian devil targeted SNP panels were created using low-coverage WGR (10–15×) data from 7–12 individuals aligned against the annotated reference genome [70,78]. A major limitation of using this low-coverage resequencing strategy is that genome regions with lower coverage can often contain sequencing errors that may not be distinguished from true SNPs [106]. This led to a number of the SNPs identified in the Tasmanian devil resequencing data not being present in the downstream SNP panel data [70,78]. While the best way to overcome this limitation is to increase the sequencing coverage of individuals, other methods, such as calling SNPs across individuals, can assist in more accurate variant calling in low-coverage WGR datasets [107].

Higher-coverage sequence data enables variants and heterozygosity to be called much more accurately than low-coverage sequence data and hence allows for SNPs to be called more confidently without additional targeted sequencing (e.g., SNP panels) [108]. High-coverage (~45×) WGR of 25 Tasmanian devils has allowed for reliable estimates of genome-wide heterozygosity, which are being used to assess the accuracy of estimates from other techniques including microsatellites, SNP panels and RRS data. The higher cost of high-coverage data causes a trade-off between investigating the whole genome of a relatively small number of individuals versus using a targeted subset of loci across many individuals (as of 2019, WGR routinely costs over $1000 per individual whereas RRS costs less than $100 per individual). This trade-off needs to be acknowledged, is dependent on the conservation research questions, and requires careful consideration prior to the commencement of sequencing [13]. Fortunately, a number of alternative cost-effective WGR approaches are available and may be suitable when high-coverage WGR is not possible. For a review of the different types of WGR and their different applications in conservation [15].

Whilst targeted sequencing approaches are useful for the exploration of genes known to be important to species biology, sometimes genetic mechanisms driving particular phenomena that are vital to species adaptation and survival may not be known or detected in other reduced sequencing techniques like RRS [109]. Whole-genome resequencing (WGR) enables conservation researchers to ask and answer a wide range of questions that are not possible using other approaches. For example, WGR also enables the use of genome-wide association studies to determine the genetic basis of particular phenotypic traits that are important to species conservation [13,15]. In the case of the Tasmanian devil, some individuals have been found to display a resistant phenotype to DFTD, enabling spontaneous tumor regression [110]. Identifying the potential genetic basis of this phenotype is important to understanding which individuals may be more resilient to the disease and provide targets for the development of potential treatments [76,77,78] (Table 1). Low-coverage WGR of individuals showing tumor regression and those that succumbed to the disease enabled a genome-wide association study to be undertaken, which identified two genomic regions that may be associated with resistance to DFTD including PAX3 and TLL1 loci [78]. A follow up study, Wright et al. [78] resequenced 10 individuals to a higher coverage (20–30×) and was able to identify a larger number of genomic regions that may underlie tumor regression in the Tasmanian devil [100]. This work demonstrates the ability of WGR data, along with an annotated reference genome, in exploring the genetic basis of phenotypic traits that could have important conservation implications [13,15,78,100] (Table 1). It is important to note that often larger numbers of individuals are required to identify genes underlying certain phenotypes, particularly in species with higher genetic diversity and reduced selective pressure on the phenotype of interest [111]. This requires careful consideration of trade-offs between the sequencing approach (targeted vs. RRS vs. WGR), number of samples and sequencing coverage, and will often depend upon some prior knowledge (or preliminary testing), budget, and access to samples. Overall, WGR data is better able to separate out and compare functional versus non-functional diversity than RRS methods, which is valuable in understanding the adaptive potential of species [94].

There are many other advantages of using this high-resolution genomic data,, including i) more robust insights into the evolutionary and demographic histories of a species; ii) more accurate measures of diversity, inbreeding and population structure; and iii) the ability to identify and investigate signatures of selection and adaptive genetic variation [15,16,18]. WGR data in the Tasmanian devil is currently being employed to assess selection and mutation rates within populations and in identifying runs of homozygosity (ROH) throughout the genome (for examples in other species, see Ceballos, et al. [112] and Hodgkinson, et al. [113]). These analyses are useful in the investigation of well-known issues in conservation, including inbreeding depression [112] and adaptation to captivity [114]. 

Some of the current limitations for using WGR in conservation contexts are the cost, the required computing power and respective expertise, and the availability of reference genomes [13,15]. Costs vary greatly and depend on the number of individuals or loci you wish to use, and the required depth of sequencing [15]. In addition, this approach requires significant expertise and compute power to execute, which limits its applicability to many conservation contexts [15]. Creating partnerships between academic researchers with the required expertise and computing resources and conservation managers is key to overcoming many limitations of using genomics in conservation, and has been successfully implemented in the conservation of the Tasmanian devil [35]. A reference genome is essential for WGR, so the significant lack of published genomes (<1%) for threatened species (or their closely-related counterparts) prevents many conservation managers from taking full advantage of high-resolution genomic data. However, in the dawn of large genomic consortia such as the Earth Biogenome Project, which aims to sequence the genomes of all of the Earth’s eukaryotic biodiversity over the next 10 years [20], lack of a reference genome will soon become a thing of the past. 

Overall, WGR paired with an annotated reference genome opens up a realm of possibilities for downstream conservation research by developing more cost-effective approaches when data from a large number of individuals is necessary for making informed conservation management decisions. As costs of sequencing continue to decrease, and the availability of reference genomes continue to rise, the use of this high-resolution genomic data in conservation research will likely become the norm [12] and is already being applied to some bird species [95].

## 4. Reference Genome Quality

An important factor to consider in the creation of reference genomes is the quality of the assembly. Consortia such as the Vertebrate Genome Project and the Earth Biogenome Project have proposed specific standards that reference genomes should meet [20,26] (Table S1). However, it is important to understand whether such high standards are necessary or achievable for conservation management. A number of statistics are used to evaluate the different aspects of genome quality including accuracy (e.g., average read coverage and quality), continuity (e.g., N50, N90, number of contigs/scaffolds, average length of contigs/scaffolds, gap percentage, etc.), and completeness (e.g., BUSCO (Benchmarking sets of Universal Single-Copy Orthologs)/CEGMA (Core Eukaryotic Genes Mapping Approach) scores, number of genes, etc.) (see Wajid and Serpedin [115] for a more exhaustive list). While the ideal reference genome would consist of a completely annotated, gap-free, chromosome-length assembly, even the some of the best model species genomes, such as the human genome, currently do not reach this standard. Furthermore, the ease and ability to reach chosen standards depends on many factors, including genome size, genome structure (e.g., repetitive content), level of heterozygosity, sample availability/quantity, as well as the cost and expertise of the sequencing types and computing resources available [24] (for reviews on reference genome creation including available sequencing types and their associated advantages/disadvantages see Ekblom and Wolf [96], Wajid and Serpedin [115], and Sedlazeck, et al. [116]). It is important to note that the current Tasmanian devil reference genome was sequenced in 2011 by Murchison et al. [46], so it does not meet the minimum standards set by the EBP (Earth Biogenome Project) or VGP (Vertebrate Genomes Project) (Table S1). Despite this, the Tasmanian devil genome has still been able to facilitate an enormous amount of conservation research. A higher-quality genome which is more complete, correct, and contiguous, has a number of advantages such as improved identification and characterization of genes and other genomic regions; more accurate ROH (runs of homozygosity) analysis and structural variant analysis; and higher resolution of chromosomal organization allowing for improved comparative genomic and evolutionary analyses [117]. 

Naturally, genome quality is also a factor of input DNA quality. High molecular weight DNA, generally greater than 40 kb in length, is required to generate the multiple sequencing types used to construct a high-quality genome [118]. Extracting high molecular weight DNA often requires additional consideration during the sample collection phase, such as flash-freezing tissues in liquid nitrogen, storage at −80 ℃ or below, and avoiding freeze-thaw. However, for species of high conservation concern, or those that inhabit difficult field locations, this could be challenging. In these scenarios, researchers may utilize museum specimens. However, this can introduce additional problems associated with sample preservation and degraded DNA, which may not be suited to long-read sequencing technologies [119]. As such, the ability to collect, store, and extract high-quality DNA should not be underestimated, as this is an essential first step towards generating high-quality genome. However, it is important to weigh up whether the cost, computing resources, expertise, and time of creating an improved or “Gold standard” assembly is necessary to answer the conservation research questions at hand. For example, Patton et al. [47] showed that the improvement of contiguity of the newly released 2019 Tasmanian devil assembly had minimal impacts on inferred patterns of historical effective population size when compared to the current reference assembly. Hence, in many cases, a simple short-read genome assembly is enough to answer many basic conservation management questions and also enable a number of more in-depth species-specific analyses mentioned in the sections above. Nevertheless, as sequencing technologies and computational infrastructure continue to advance and become more affordable, high-quality reference genomes would become easier to create and would overcome many of the limitations of currently fragmented reference assemblies such as incomplete gene characterization, comparative evolutionary limitations, and increased computational requirements [117]. Despite this, without advances in sequencing chemistry and library preparation to reduce input DNA quality and quantity, the availability of high-quality samples and ensuing high molecular weight DNA may continue to limit the creation of high-quality reference genomes in some species.

## 5. Conclusions

The Tasmanian devil reference genome has enhanced our capacity to manage this species in the face of an infectious, clonal cancer. By having the reference genome, we have been able to develop a range of genomic tools that have been used to investigate DFTD (e.g., [46]), investigate the interplay between the Tasmanian devils and the disease (e.g., [76,77,78,79]), inform development of immunotherapy and vaccine protocols [66], inform the management of the insurance population [38,65], and provide advice on the translocation of Tasmanian devils to wild populations to improve both genome-wide and functional diversity (e.g., [63,89]). Tasmanian devils are not the only species who are threatened globally by disease; other examples include black-footed ferret and distemper [120], bats and white-nose syndrome [121], and frogs and chytrid [122]. Here we have presented a strong case study of the benefits of using reference genomes for the conservation of threatened species. As the threat to global biodiversity increases, the management of threatened species becomes more pronounced. Reference genomes could be used by conservation managers to develop a range of genetic tools such as designing species-specific microsatellite markers for population data and differentiation; developing targeted SNP panels, or aligning and calling RRS data, for higher resolution population information or data on particular genes of interest; and conducting exploratory analyses (e.g., genome-wide association studies) using variant calling of whole-genome resequencing data. 

Despite the challenges in obtaining high-quality samples for genome sequencing and expertise for the creation of reference genomes for threatened species, there is value in them. Reduced costs and lower input DNA requirements, as well as improved bioinformatic assembly and annotation pipelines based on non-model non-eutherian species, mean that these technologies are becoming more attainable by conservation programs and should be used more routinely where budgets allow [96]. Reference genomes enable a wealth of genetic/genomic applications and are an important asset in our ongoing fight to preserve global biodiversity. We would recommend that conservation managers who are seeking to use the types of methods we have described herein collaborate with global genome consortia (like the Earth Biogenome Project) or national/local consortia (like the Oz Mammal Genome Initiative) to utilize the full potential of genomic resources and join the genomics revolution. This allows conservation managers to focus on conservation and work with geneticists who can help them make adaptive management decisions in real-time [35].

Although here we have presented a unique case study of a species with significantly low levels of genetic diversity and a large threatening disease process, the techniques described for the Tasmanian devil can be applied more broadly to many species of conservation concern. The applications of what we have described herein for devils is not unique to this species as many of the questions we have answered are posed by those managing other threatened species. These include understanding historical demography and current population structure, minimizing inbreeding, maximizing adaptive potential, and identifying the basis of important phenotypic traits (whether these be related to disease, behavior, or reproduction). Hence, despite differences in threatening processes and current state of vulnerable species, the nature of their small population sizes will result in a number of common conservation concerns that could be informed using genomic data [15,18]. In the midst of the sixth mass extinction event, we advocate the use of reference genomes and associated genetic tools to arm conservation managers with ways to assist the long-term survival of species.

## Figures and Tables

**Table 1:** Examples of Tasmanian devil conservation questions, actions, and outcomes that have been facilitated by the reference genome.

Reference Genome Use	Conservation Questions Addressed	Conservation Actions	Conservation Outcomes
• Microsatellite development • Genome-wide SNP analysis	• Were the founders related? • Does the metapopulation have equal founder representation to ensure the maintenance of gene diversity? • Is inbreeding accumulating in group housing and Maria island insurance populations?	• Resolved relatedness of founders [43] • Resolved parentage in group housing within the metapopulation [50,52,54] • Reconstructed pedigree of island population [53] • Informed translocation recommendations [63]	• Tool for selecting individuals for translocations based on genetic complementation • Improved maintenance of genetic diversity across captive populations • Increased genetic diversity of hybrid individuals at wild release sites
• The characterization of DFTD strains	• How many DFTD strains exist?	• Appropriate management of wild populations [46,64,65]	• Assisted in managing the spread of new DFTD strains
• The characterization of immune genes • Primer design and SNP panel development • Targeted SNP analysis	• Can we develop a vaccine for DFTD? • Can we improve Tasmanian devil immune diversity?	• Immunization development and deployment [66]. Immune gene diversity analysis for informed translocation recommendations [67,68,69,70,71,72,73,74,75]	• Improved immune responses of devils released to the wild • Improved immunogenetic diversity of released Tasmanian devils and their resultant offspring
• Development of blocking primer for metagenomics diet analysis	• What constitutes the complete diet of Tasmanian devils on Maria Island?	• Investigating the impact of an introduced carnivore to island wildlife	• Mitigation implemented to reduce the impact on highly consumed species
• Alignment of resequenced genomes • SNP Analysis and Annotation • GWAS	• Are devils evolving host-parasite resistance to DFTD?	• Ongoing monitoring to ensure releases do not impact the evolution of potential resistance alleles [76,77,78,79]	• Assisted in understanding regions of the genome that are potentially involved in DFTD resistance

## Supplementary Materials

The following are available online at https://www.mdpi.com/2073-4425/10/11/846/s1, Table S1: Comparison of model and non-model mammalian/marsupial reference genomes to the G10K and EBP minimum reference genome quality standards.
