# Supplementary material for: The Value of Reference Genomes in the Conservation of Threatened Species
Source: Genes (Basel). 2019 Oct 25;10(11):846. doi: 10.3390/genes10110846 (PMC6895880; doi:10.3390/genes10110846)
Supplement: Supplementary file 1 [file genes-10-00846-s001.pdf]

Supplementary Material

The value of reference genomes in the conservation of threatened species

Parice Brandies[1], Emma Peel[1], Carolyn J. Hogg[1], Katherine Belov[1]*

[1] School of Life & Environmental Sciences, The University of Sydney, Sydney, Australia
* Correspondence: kathy.belov@sydney.edu.au

Table S1. Comparison of model and non-model mammalian/marsupial reference genomes to the G10K and EBP minimum reference genome quality standards. Green: metrics matching the G10K standards, Yellow: metrics matching the EBP Phase I standards, RED: metrics matching the EBP Phase II standards, Grey: metrics which fall below all VGP and EBP standards.

Reference Genome Minimum Quality Standards

Project	Phase	Contig N50	Scaffold N50	% Genome assembled into chromosomes	Inter-chromosomal rearrangements validated by >2 data sources	QV Cut-off Score*	Genome Quality Metric^
G10K	-	1Mb	10Mb	>90%	Yes	40	3.4.2.QV40
EBP	I	0.1Mb	1Mb	>90%	Yes	40	2.3.2QV40
EBP	II	0.01Mb	0.1Mb	>90%	No	40	1.2.1QV40

Current Mammalian/Marsupial Reference Genome Metrics

Species	Genome	Contig N50 (Mb)	Scaffold N50 (Mb)	% Genome assembled into chromosomes	Inter-chromosomal rearrangements validated by >2 data sources	QV Cut-off Score	Genome Quality Metric	Date Published to NCBI
Human	GRCh38.p13	57.9	67.8	99.86%	Yes	ND	4.4.2QV?	28/2/19
Mouse	GRCm38.p6	32.8	54.5	99.97%	Yes	ND	4.4.2QV?	15/9/17
Dog	CanFam3.1	0.267	45.9	96.54%	Yes	ND	2.4.2QV?	2/11/11
Koala	phaCin_unsw_v4.1	11.6	-	0.00%	No	ND	4.4.0QV?	18/4/17
Tasmanian Devil	Devil_ref v7.0	0.0201	1.85	99.96%	Yes	30	1.3.2QV30	17/2/11

* ND = Not Determined.

^ The genome quality metric summarises all of the minimum standards from the previous columns whereby the first three numbers are the exponents of the N50 contig, N50 scaffold and level of chromosomal assembly and QV represents the minimum base-call quality error.
